# Behavior of Dolomites under Fluidized Bed Sorption-Enhanced Gasification Conditions

**DOI:** 10.1021/acs.iecr.4c04520

**Published:** 2025-02-27

**Authors:** Antonio Coppola, Fiorella Massa, Fabrizio Scala, Fabio Montagnaro

**Affiliations:** † Institute of Sciences and Technologies for Sustainable Energy and Mobility (STEMS), National Research Council (CNR), Piazzale Vincenzo Tecchio 80, 80125 Naples, Italy; ‡ Department of Chemical, Materials and Industrial Production Engineering, University of Naples Federico II, Piazzale Vincenzo Tecchio 80, 80125 Naples, Italy; § Faculty of Electrical Engineering and Computer Science, VŠB-Technical University of Ostrava, 17. Listopadu 2172/15, Poruba, Ostrava 708 00, Czech Republic; ∥ Department of Chemical Sciences, University of Naples Federico II, Complesso Universitario di Monte Sant’Angelo, 80126 Naples, Italy

## Abstract

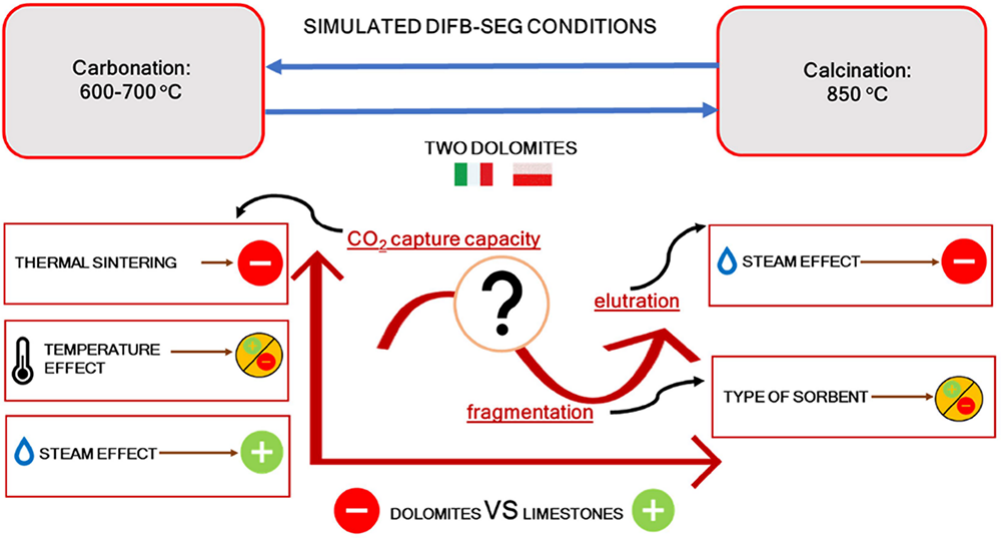

This work investigates the behavior of dolomites during sorption-enhanced gasification in dual interconnected fluidized bed systems for the production of H_2_-rich syngas. Two sorbents, one of Italian origin, the other of Polish origin, with CaO content ranging from 60 to 63 wt %, and MgO content around 32–33 wt %, were subjected to calcination/carbonation cycles (10 cycles for each test) in a lab-scale apparatus. In particular, the temperature of the fluidized bed carbonation stage was varied between 600 and 700 °C, both in the absence and in the presence of water vapor. With an eye to the role of the operating conditions, for each material, the following aspects were investigated: CO_2_ capture capacity, fragmentation tendency, and elutriation phenomena upon surface wear. The discussion was then extended by recalling previous results obtained on a set of 6 limestones (rich in Ca) under the same operating conditions through a comparison analysis between the behavior of dolomites versus limestones. In particular, it was observed that steam is capable of positively influencing the dolomite behavior in terms of CO_2_ capture; the elutriation rate is markedly more relevant upon calcination than carbonation, negatively affected by the presence of steam, and less extensive for sorbents showing higher CO_2_ capture. Normalizing the CO_2_ capture on the CaO content, the performance of dolomites is comparable to that of limestones, although dolomites have a more fragile structure that increases their tendency to undergo fluidized bed fragmentation.

## 1 Introduction

**Figure 1:**
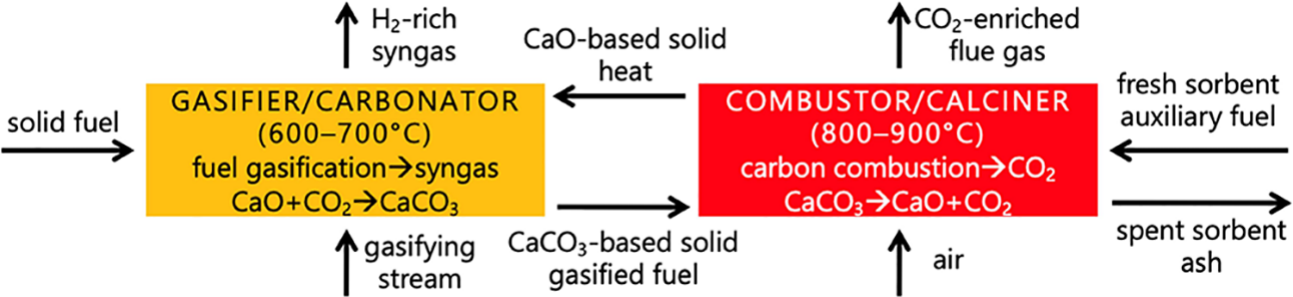
DIFB-SEG simplified scheme.

Upon gasification of a solid fuel, e.g., of a biomass, the main constituents of the syngas (H_2_, CO, CO_2_) can interact with each other, in the presence of H_2_O, according to the homogeneous equilibrium reaction of Water–Gas Shift (WGS): CO + H_2_O = CO_2_ + H_2_. In Sorption-Enhanced Gasification (SEG),^1−7^ a stream of CaO is fed to the system (originating from a fresh sorbent such as limestone). In this case, the gasifier also takes on the role of a carbonator, as schematically depicted in Figure 1. In fact, CaO is able to capture CO_2_ thanks to the heterogeneous carbonation reaction, thus subtracting CO_2_ from the reaction environment. This allows the WGS reaction to determine an enhanced formation of H_2_ which, as promoted by the CO_2_ sorption, gives the name to the process (SEG). The calcium-based sorbent stream, once carbonated, is sent to a second reactor, where the calcination reaction takes place (the reverse of carbonation): CaO is regenerated, ready for another cycle. At the same time, the flue gas leaving this stage will be enriched in CO_2_, but only additional techniques (e.g., operating the reactor in oxyfuel mode, or postprocessing the flue gas) would enable the recovery of CO_2_ at concentration levels compatible with its geological storage or utilization.

Borrowing the concept of “calcium looping”,^8^ SEG can be operated in a system consisting of Dual Interconnected Fluidized Beds (DIFB), given the need to circulate solid sorbent particles from one reactor to the other (Figure 1). It should also be remembered that the two fluidized beds will work at different temperatures since the carbonation process is exothermic and the calcination process is endothermic. Therefore, CaO exiting the calciner (at a higher temperature) will also be able to act as a heat carrier at the inlet of the carbonator to sustain the gasification reaction.

This study focuses on the behavior of sorbent particles under repeated calcination/carbonation cycles, in conditions resembling those of DIFB-SEG. This analysis is important for the correct design of the process since, following multiple calcination/carbonation cycles, thermal sintering effects induce a decrease in the CO_2_ capture capacity, the latter representing an essential parameter to allow the desired H_2_ yields in the gasifier. Furthermore, the operation of FB reactors is associated with attrition and fragmentation phenomena of the sorbent particles,^9−14^ with elutriation of fines and alteration of the particle size distribution in the reactor (and, therefore, of the distribution of residence times). These aspects are also essential to design the necessary makeup of the sorbent to be sent to the system, to compensate for both chemical (sintering) and physical (elutriation) losses of material.

The analysis was conducted considering two aspects that do not represent the benchmark for the majority of literature works on the subject: (i) the conduction of tests in a lab-scale DIFB system and (ii) the focus on dolomitic sorbent materials. In relation to the second point, in fact, reference is often made to limestone-based sorbents, therefore rich in CaCO_3_, as the source of reactive CaO. Dolomites, natural materials based on CaCO_3_ and MgCO_3_,^12^ can represent an alternative in contexts where the use of this sorbent is more convenient, but they have been little studied for fluidized bed SEG applications, although the concept of using dolomite-based sorbents has been reported by, e.g., Santos and Hanak.^15^ If dolomite is used as a natural fresh sorbent instead of limestone, first the two carbonates are converted into the respective oxides (CaCO_3_ → CaO+CO_2_ and MgCO_3_ → MgO+CO_2_). Then, upon cycling, while MgO remains substantially inert from the chemical point of view, CaO takes part into the looping process as described above.

Our previous works^16−18^ were focused on a set of 6 commercial limestone-based sorbents, analyzing the effect of steam (which is present in a gasification environment) and temperature during the carbonation stages. These analyses are here extended, to integrate the knowledge framework, also to 2 dolomitic sorbents. In particular, the following aspects were investigated: CO_2_ capture capacity, fragmentation tendency and elutriation phenomena upon surface wear. A comparison analysis between the behavior of limestones (from previous work) and that of dolomites complemented the study.

## 2 Experimental Section

### 2.1 Dolomite Sorbents and Dual Interconnected Fluidized Bed Reactor

The two dolomites used in this study, whose chemical composition in terms of oxides as obtained by X-ray Fluorescence (XRF) is reported in Table 1, are DOM-I (an Italian sorbent, slightly richer in Ca) and DOM-R (a Polish sorbent, where “R” stands for Rędziny, Upper Silesian region, slightly richer in Si). The Mg-content is, instead, comparable.

**Table 1:** Chemical Composition (in Terms of Oxides, wt %) of the Two Dolomites

	CaO	MgO	SiO_2_	Al_2_O_3_	SO_3_	Fe_2_O_3_	K_2_O	MnO	total
DOL-I	63.1	32.5	1.95	1.47	0.489	0.308	0.151		99.968
DOL-R	59.8	32.7	3.98	1.77	0.396	0.746	0.189	0.382	99.963

Data in Table 1 can be reworked by assuming the parent dolomites composed by CaCO_3_ and MgCO_3_ (from which the composition of CaO and MgO is obtained by XRF), plus other noncarbonated oxides. By simple mass balances, the mass fraction of CaCO_3_ results as *f*_CaCO_3__ = 0.608 for DOL-I and *f*_CaCO_3__ = 0.584 for DOL-R.

**Figure 2:**
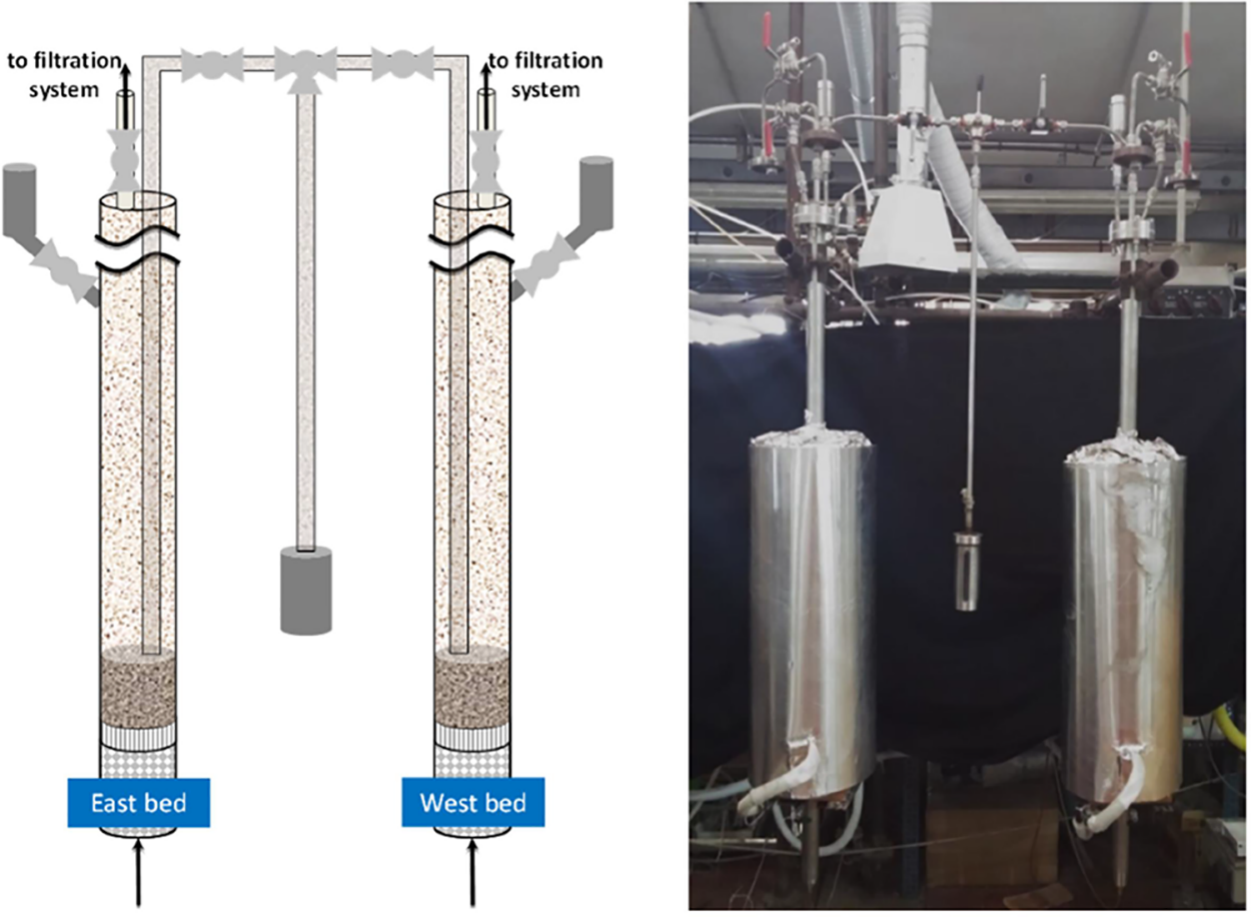
“Twin Bed” DIFB reactor for SEG tests (left: scheme; right: picture).

SEG tests were carried out in a lab-scale DIFB system, as illustrated in Figure 2. It is based on two identical 40 mm-ID fluidized beds (both operated in a bubbling regime). The two reactors (one acting as the calciner and the other as the carbonator) are electrically heated, and they are connected to each other by a 10 mm-ID duct which is in part immersed in the FB dense zone, allowing the pneumatic transport of the solid material from one reactor to the other (the reactors are batchwise operated with respect to solid). Further details are retrievable in our previous works.^19,20^ Moreover, the exit section of each reactor is equipped with an exhaust duct whence the flue gas is conveyed to sintered steel filters (efficiency >99% for >10 μm particles) for collection of elutriated fines.

### 2.2 Operating Conditions of Experimental Tests

Table 2 shows the main operating conditions for the DIFB-SEG tests. Gas cylinders, equipped with a flow meter/regulator, were used to obtain surrogate gases so as to simulate SEG conditions. Moreover, steam was produced (at temperature *T* = 200 °C) via a generator connected to a liquid-flow system (Bronkhorst) equipped with a flow meter.

**Table 2:** Main Operating Conditions for the DIFB Tests

stage	*T*, °C	duration (min)	vol.% CO_2_	vol.% H_2_O (balance)
calcination	850	10	10	0 (air)
dry carbonation	600/650/700	10	10	0 (N_2_)
wet carbonation	600/650/700	10	10	10 (N_2_)

The tests, on each of the two dolomite sorbents (presieved in the 400–600 μm size range), consisted in 10 complete calcination/carbonation cycles, plus an 11th calcination stage (for a total of 21 stages). At the beginning of a test, an initial mass of sorbent of *m*_0_ = 10 g was charged into the calciner. For both reactors, we used 800–1000 μm silica sand as the fluidization/thermal ballast material. An amount of approximately 150 g of sand was present in each reactor. In dedicated preliminary tests, we verified the sand’s negligible tendency to fragmentation; therefore, the size of the sand particles was chosen sufficiently coarser than that of the dolomitic sorbent to avoid that, at the end of the test, the granulometric characterization of the sorbent could be influenced by the presence of sand.

Calcination was carried out at *T* = 850 °C, in a fluidizing gaseous atmosphere composed by 10% CO_2_ by volume (balance, air), to simulate oxidizing conditions in an air-fed combustor-calciner. Carbonation was carried out under, again, 10% CO_2_, choosing, for each sorbent, 6 different operating conditions: 3 temperature levels (600, 650, 700 °C), each in the absence (dry carbonation) or presence (wet carbonation) of steam. When present, steam concentration was 10%. Under carbonation, the balance gas was N_2_ to simulate the reducing conditions typical of the gasifier-carbonator.

Each stage lasted 10 min, so as to observe the practical completion of the reactive processes (either calcination or carbonation). Both beds were fluidized at a superficial velocity of 0.5 m/s under process conditions, which is slightly more than twice the average minimum fluidization velocity for the bed material. This value optimized the desired segregation between the dolomite sorbent and bed material, which is important to obtain a good pneumatic sorbent transport between the two reactors while minimizing sand transfer. In particular, under the process conditions, the minimum fluidization velocity of the sand particles was 0.22 m/s, and that of the dolomite particles was 0.055 m/s. Regarding the terminal velocity of the solids, it was approximately 3.3 and 8.2 m/s for dolomite and sand particles, respectively.

### 2.3 Parameters under Investigation

The CO_2_ concentration in the gas stream exiting the carbonator was continuously monitored via an NDIR analyzer (ABB Advance Optima AO2020). This allowed us to calculate the sorbent CO_2_ capture capacity, defined as the mass of CO_2_ captured in the *N*th carbonation stage per initial mass of sorbent:
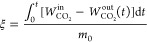
1where *W*_CO_2__^in^ is the mass flow rate of CO_2_ at the carbonator inlet, *W*_CO_2__^out^(*t*) is the mass flow rate of CO_2_ at its exit, and *t* is the carbonation time. We will also refer to the degree of carbonation of Ca (the moles of Ca that reacted cumulatively toward CaCO_3_ in the generic *N*th stage of carbonation, divided by the moles of Ca initially loaded into the system), under the assumption that CO_2_ is captured by CaO (cf. Figure 1):
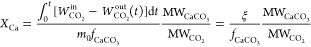
2where MW stands for molecular weight.

The Particle Size Distribution (PSD) of the sorbent was characterized by manual sieving of the bed material at the end of the tests. Sieving was carried out in 8 size intervals, from 0–112 to 400–600 μm, with an average diameter, *d*_*i*_, thus varying between 56 μm (0–112 μm interval) and 500 μm (400–600 μm interval). We define *x*(*d*_*i*_) as the mass fraction of particles that fall into a size range having an average diameter *d*_*i*_. Moreover, we define particles finer than the lower limit of the initial particle size range (400–600 μm) as “fragments”. Therefore, the cumulative mass fraction of fragments is
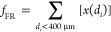
3

Elutriation (by attrition) of the dolomite sorbents was characterized by working out the mass of fines elutriated at the exhaust and collected in the filters. The specific elutriation rate *E* was defined as the mass of sorbent fines cumulatively collected over a given stage, *m*_el_, divided by the initial mass of sorbent and by the duration of the stage itself, Δ*t* (=10 min):
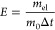
4

Elutriation data can be equivalently expressed in terms of the mass fraction of fines lost by elutriation:
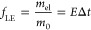
5

## 3 Results and Discussion

Results will be now discussed with reference to the 12 investigated cases. The labeling is DOL-*X*/*T*°C/*Z*, where *X* = I or R for DOL-I or DOL-R, respectively; *T* is the carbonation temperature; *Z* = DRY or WET if carbonation is carried out in the absence or presence of steam, respectively.

### 3.1 CO_2_ Capture Capacity of Dolomite Sorbents

**Figure 3:**
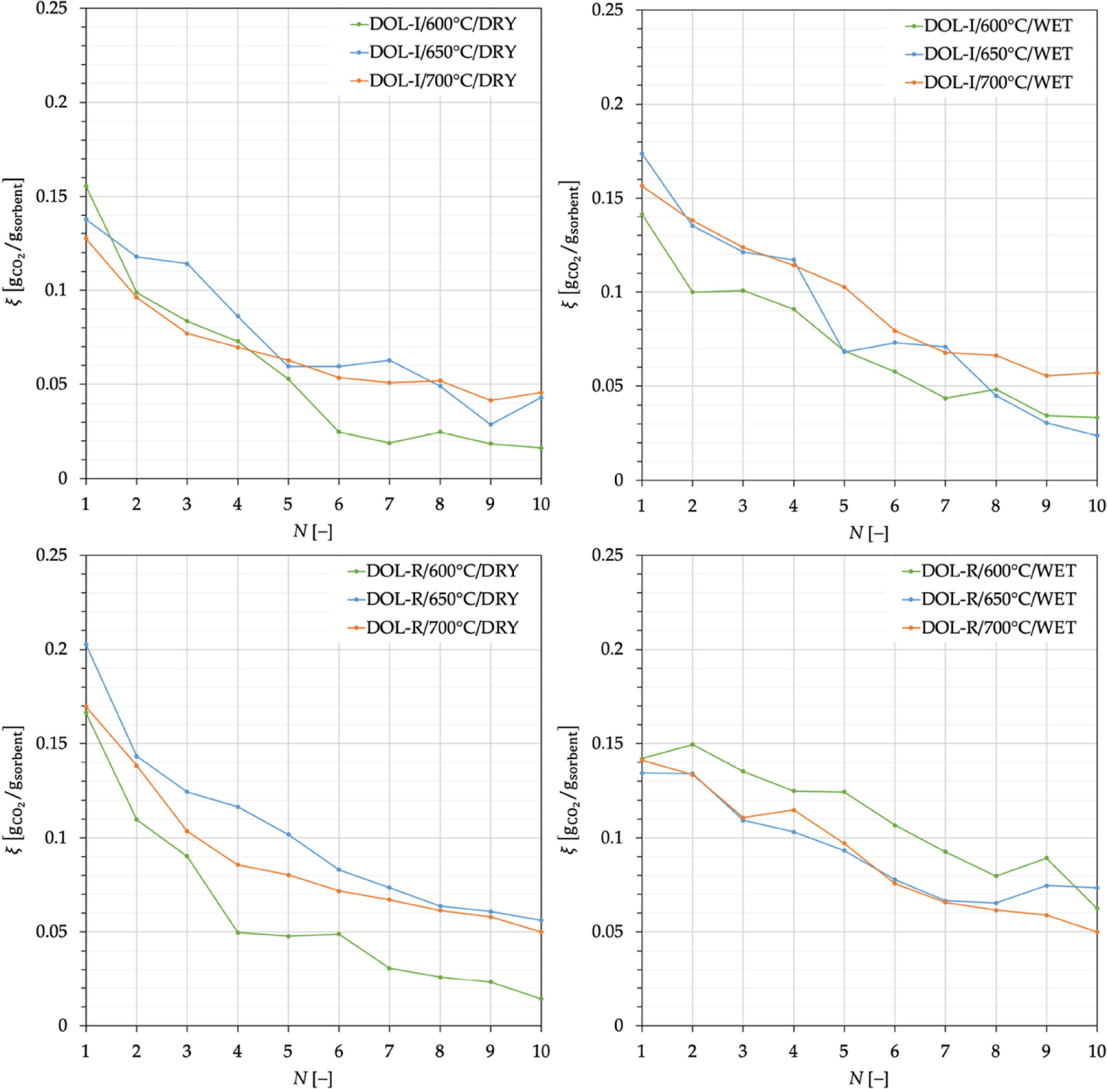
CO_2_ capture capacity, eq 1, for DOL-I and DOL-R as a function of the number of carbonation stages carried out at temperature of 600, 650, and 700 °C, in the absence (dry conditions) and presence (wet conditions) of steam.

Trends of ξ(*N*), eq 1, are reported in Figure 3 for all of the investigated conditions. In each of the 12 cases, the decay of ξ along *N* can be observed, which is related to the effect of thermal sintering that limits the sorbent performance upon iterated calcination/carbonation cycles. Values of the CO_2_ capture capacity range between 0.127–0.203 g_CO_2__/g_sorbent_ after *N* = 1, down to 0.014–0.073 g_CO_2__/g_sorbent_ after *N* = 10. In percentage terms, the loss of CO_2_ capture capacity between the first and the 10th cycle is in the range 45–92%. In addition, a residual activity of the sorbents at the 10th cycle can be observed, although constraints related to the operation of the DIFB system did not allow analysis of the behavior of the materials in the very long-term.

**Figure 4:**
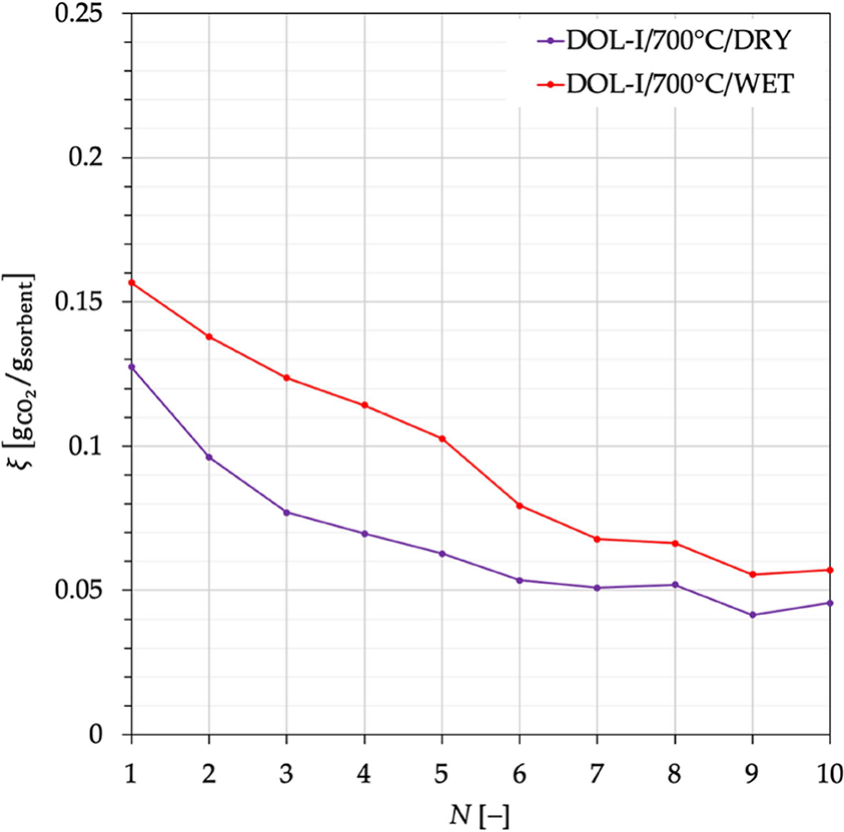
Effect on the CO_2_ capture capacity of the presence of steam during carbonation at 700 °C for DOL-I.

Table 3 reports the average value for the CO_2_ capture capacity (ξ_av_) over the *N* = 10 carbonation stages. The following can be observed as general trends:

DOL-R appears slightly more reactive than DOL-I, although the content of CaO is, for DOL-R, lower (cf. Table 1). The reasons behind this behavior should be sought in the different porous structure of the materials, probably more favorable for DOL-R, and can be the subject of future investigations, possibly complemented by chemical analyses on the main species (CaO, MgO, CaCO_3_) constituting the sorbent particles along the DIFB-SEG cycles. The study of the physical/chemical changes occurring to particles could, in addition, help in strengthening the relation between operating conditions and decay in CO_2_ capture capacity along the cycles;
The presence of steam upon carbonation improves the dolomites performance. An example is given in Figure 4 for DOL-I carbonated at 700 °C, with ξ_av_ increasing by 42% in the presence of steam. This is related to the enhanced diffusion of CO_2_ inside the pores of the sorbent particle, with the development of a more favorable sorbent morphology and the formation of ionic (OH^–^) species able to improve CO_2_ capture, a feature also observed for limestone-based sorbents;^18,21−23^
No unidirectional effect of carbonation temperature can be retrieved, as the following aspects might concurrently be at work when increasing *T* upon carbonation: nonmonotonic trend for the carbonation kinetics due to the exothermic nature of this (equilibrium) process; improvement of CO_2_ solid-state diffusion; increased sintering.

**Table 3:** Relevant Values of the Investigated Parameters

	DOL-IDRY	DOL-IWET	DOL-RDRY	DOL-RWET
ξ_av_(600 °C) [g_CO_2__/g_sorbent_]	0.0566	0.0719	0.0607	0.1106
ξ_av_(650 °C) [g_CO_2__/g_sorbent_]	0.0758	0.0858	0.1026	0.0932
ξ_av_(700 °C) [g_CO_2__/g_sorbent_]	0.0676	0.0961	0.0886	0.0909
*f*_FR_(600 °C) [−]	0.594	0.642	0.798	0.754
*f*_FR_(650 °C) [−]	0.596	0.619	0.757	0.736
*f*_FR_(700 °C) [−]	0.610	0.625	0.748	0.741
*f*_LE,av_(600 °C) [mg_fines_/g_sorbent_]	0.652	2.027	0.385	0.921
*f*_LE,av_(650 °C) [mg_fines_/g_sorbent_]	1.266	1.820	0.393	0.994
*f*_LE,av_(700 °C) [mg_fines_/g_sorbent_]	1.373	1.360	0.489	0.828

### 3.2 Sorbent Fragmentation and Attrition

**Figure 5:**
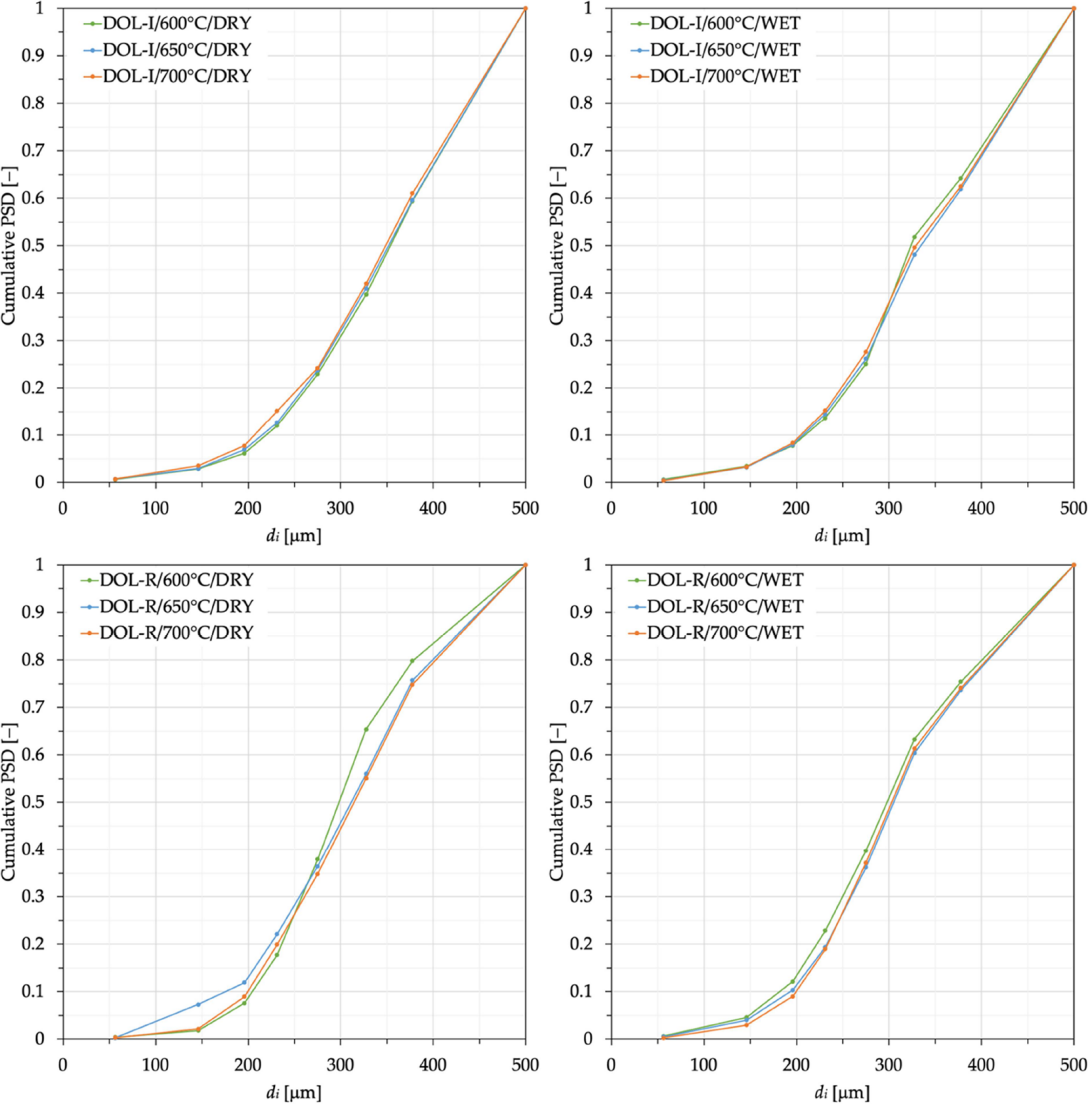
Cumulative particle size distribution for sorbent fragments retrieved at the end of the 21 DIFB-SEG stages. Data for DOL-I and DOL-R after carbonation were carried out at temperatures of 600, 650, and 700 °C, in the absence (dry conditions) and presence (wet conditions) of steam.

**Figure 6:**
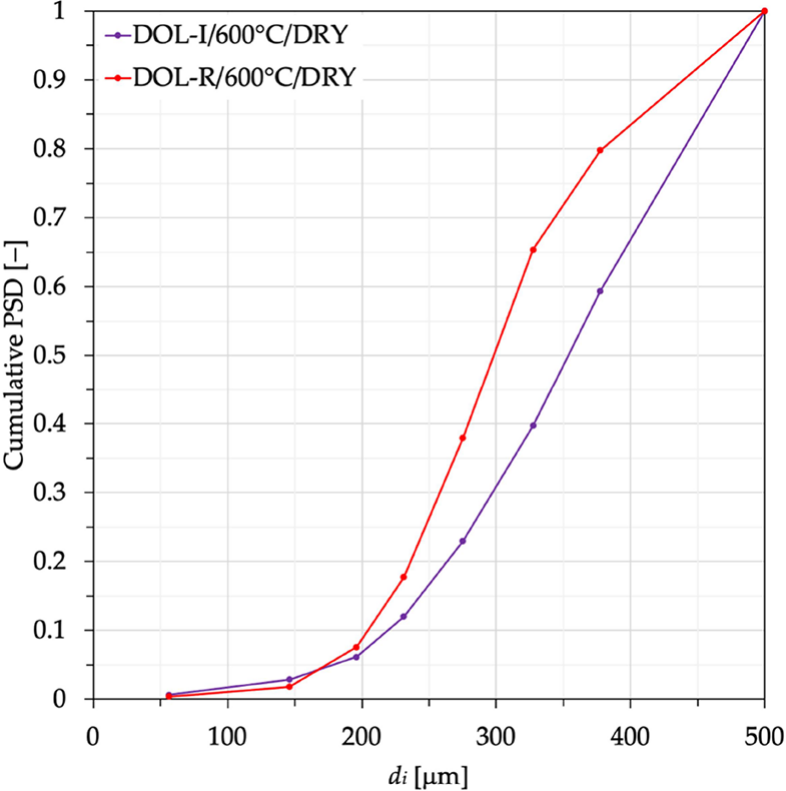
Cumulative particle size distribution for sorbent fragments retrieved at the end of the 21 DIFB-SEG stages: comparison between DOL-I and DOL-R after carbonation carried out at 600 °C in the absence of steam.

Cumulative PSD after the tests for all of the investigated cases is reported in Figure 5, while Table 3 lists the corresponding values for *f*_FR_ (eq 3). The cumulative mass fraction of fragments can be as high as 80% for DOL-R carbonated in the absence of steam at 600 °C, and it resulted not lower than 59% (the case of DOL-I under similar conditions). By taking this condition as an example of a comparison between the two sorbents, it is clearly observed in Figure 6 that DOL-R is more subject to fragmentation than DOL-I (in this case, *f*_FR_ for DOL-R is ∼35% higher).

Indeed, fragmentation of dolomites is relevant in all cases, confirming a general scenario that depicts these sorbents (both Ca- and Mg-based) more prone than ordinary limestones (Ca-based) to suffer particle breakage phenomena,^12,24−27^ as it will be discussed later on.

When fragmentation data are looked at, it has to be recalled that this phenomenon is not necessarily negative in terms of sorbent performance. In fact, this analysis pictures the particle size distribution of nonelutriated fragments, i.e., finer particles (than the parent size range) that are still able to remain in the bed and to take part in the reactive carbonation process, with possible enhanced activity due to their smaller size. With this view in mind, the larger fragmentation occurring for DOL-R could in part explain its better CO_2_ capture capacity vs DOL-I (cf. data for ξ_av_ in Table 3).

Discussing now the elutriation data, values of *E* have been reworked in terms of *f*_LE_, eq 5. Table 3 reports the average values (*f*_LE,av_) over the whole set of calcination and carbonation stages. Values for *f*_LE,av_ range between 0.385 mg_fines_/g_sorbent_ and 2.027 mg_fines_/g_sorbent_. The presence of steam is generally able to weaken the external structure of dolomite particles, yielding larger values for elutriation rates—it is, in fact, recalled that elutriation of fines mostly derives from the wear of the particles’ external surface under the effect of the bubble motion in a fluidized bed. The presence of steam can increase *f*_LE,av_ by a factor as high as 3.1 (DOL-I, dry carbonation at 600 °C). The inspection of data in Table 3 also reveals a tendency, for DOL-I, to be more prone to surface wear than DOL-R (*f*_LE,av_, at given operating conditions, increases by a factor of 1.6–3.2). This could also be related to the higher carbonation for DOL-R, with a consequently harder shell (being carbonates harder than the corresponding oxides), which makes these particles more resistant.

**Figure 7:**
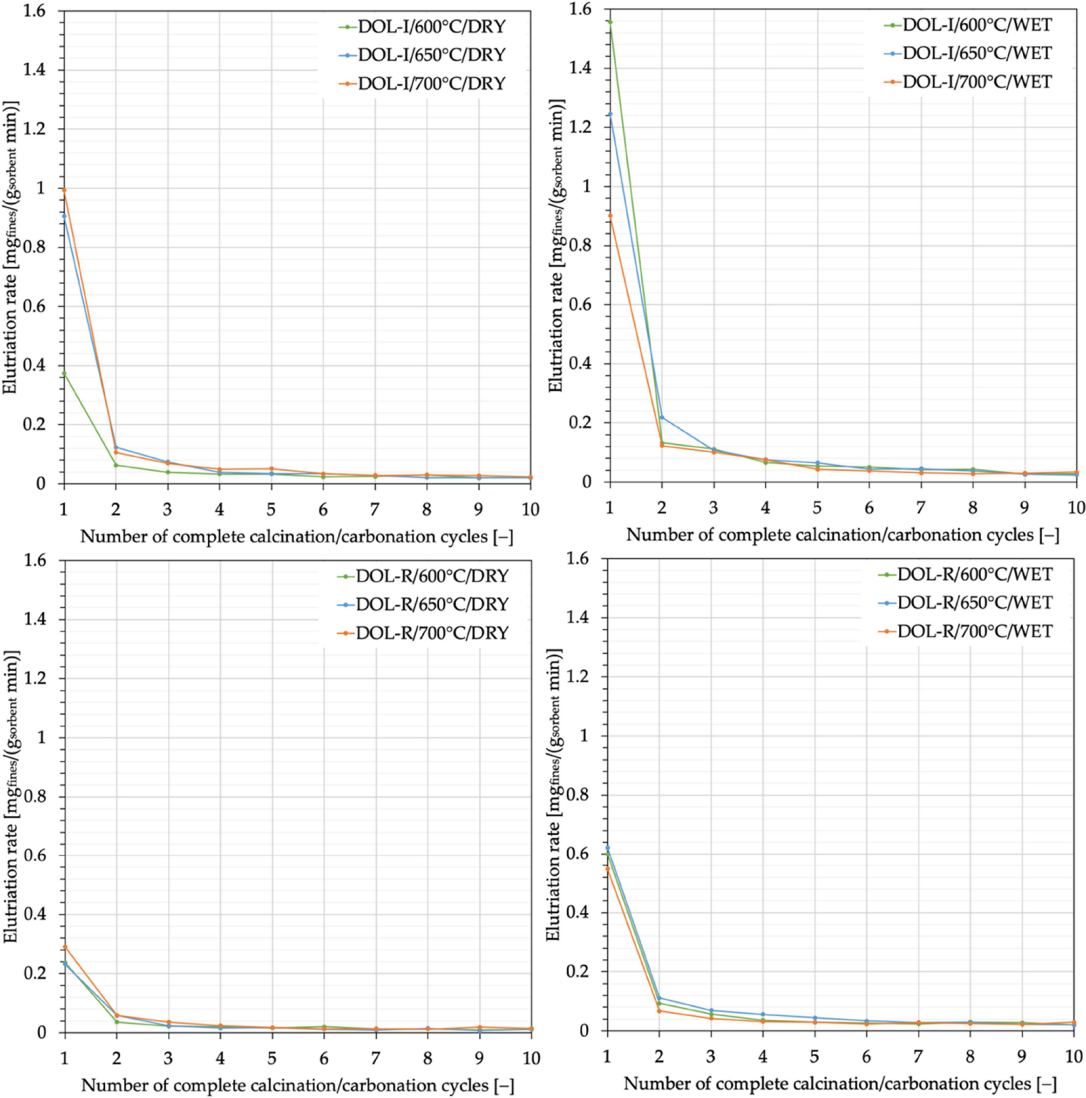
Elutriation rate (average values per cycle), eq 4, as a function of the number of looping cycles. Data for DOL-I and DOL-R after carbonation carried out at temperatures of 600, 650, and 700 °C, in the absence (dry conditions) and presence (wet conditions) of steam.

**Figure 8:**
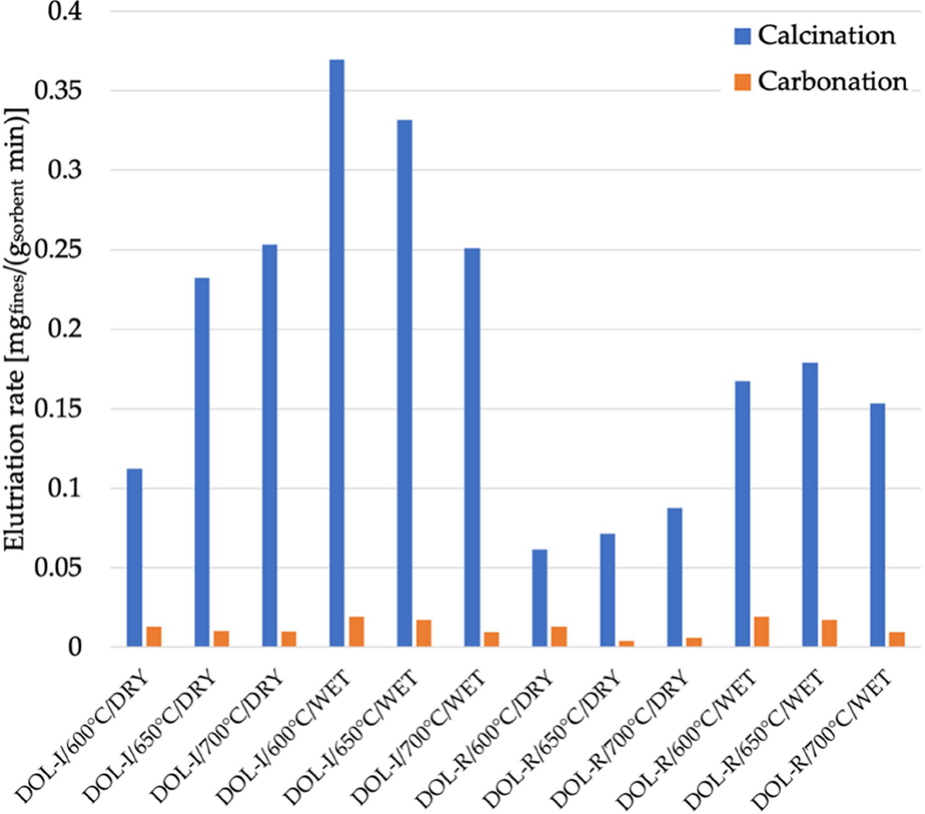
Elutriation rate averaged over the whole number of calcination stages or the whole number of carbonation stages for the entire set of operating conditions.

Further considerations on the elutriation rate are as follows:

1. The elutriation rate is a function decreasing with the number of cycles, as can be seen in Figure 7. Moreover, in the first cycles (especially in the first one), the removal of the initial surface asperities of the fresh sorbent particles takes place, determining greater elutriation. Furthermore, as the residence time in the system increases, the particles undergo thermal sintering phenomena which, although negative in terms of carbonation capacity, make them more resistant from a mechanical point of view;
2. The decrease in the elutriation rate as illustrated in Figure 7, between the first and the last cycle, is always >94%;
3. As shown in Figure 8, the elutriation rate is markedly more relevant during calcination than during carbonation (by a factor of about 5–26). This is likely due to the combined effect of the higher temperature (higher mechanical stresses) experienced by the particles in the calcination stages, and of the rapid release of CO_2_ upon calcination generating internal overpressures in the particles.

### 3.3 Comparison between Dolomites and Limestones

**Figure 9:**
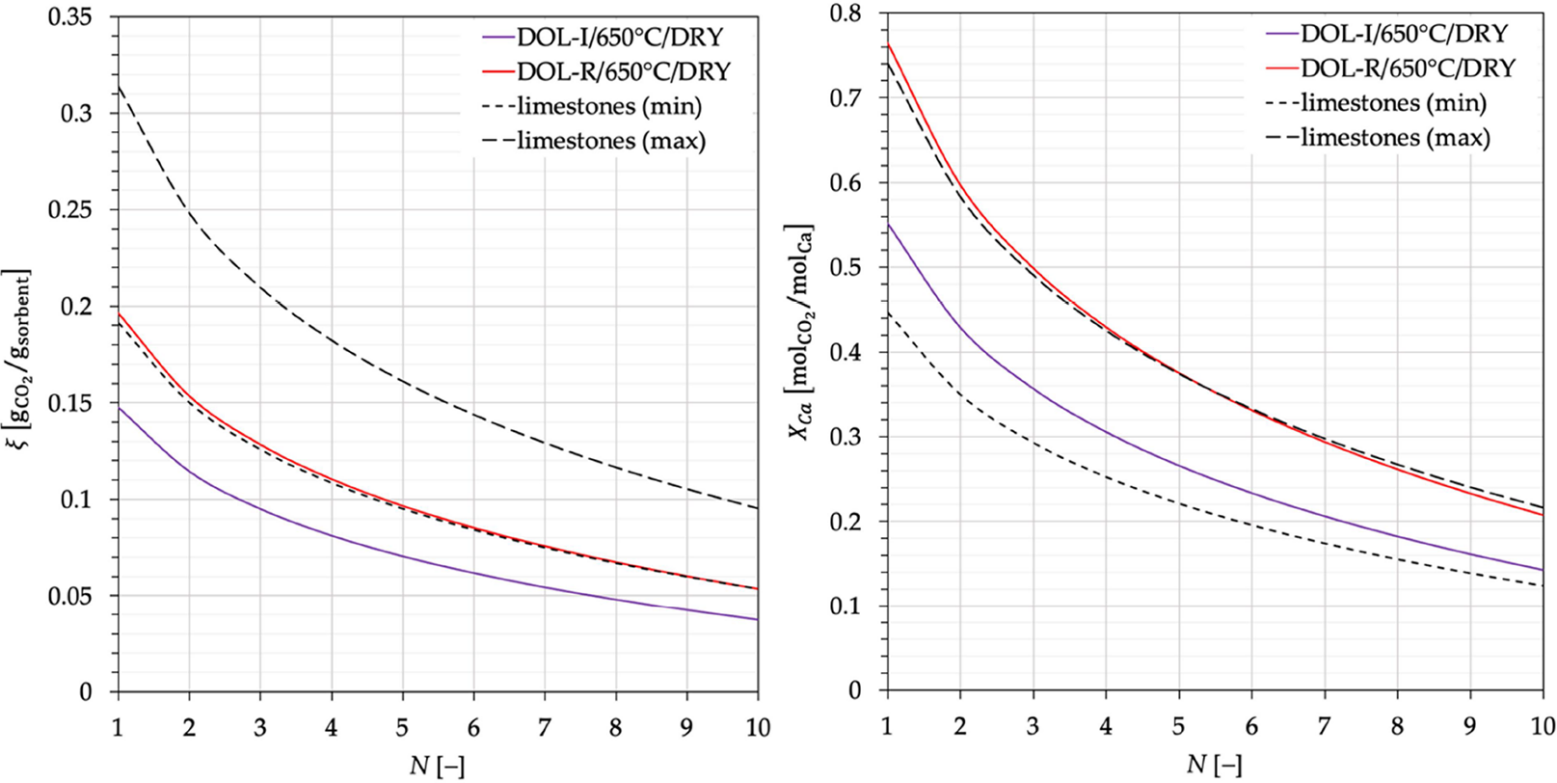
Left: CO_2_ capture capacity; Right: degree of calcium carbonation, for DOL-I and DOL-R (carbonated under dry conditions at 650 °C) as a function of the number of carbonation stages, compared with the range of values obtained, under identical conditions, for a set of 6 limestones.

The behavior observed in this work for the two dolomite-based sorbents is now compared with that observed for a set of 6 limestones tested under similar conditions, as reported in previous papers.^16−18^Figure 9(left) shows, as an example for dry carbonation at 650 °C, the CO_2_ capture capacity for the two dolomites, ξ(*N*), in comparison with the minimum and maximum values obtained for the limestones. These trends have been plotted as continuous curves (corresponding to best-fitting curves) to increase readability of the data. The lower performance of the dolomites with respect to the limestones is clearly evident (and this is also valid for all the other operating conditions): since the reactive species toward CO_2_ is only CaO (MgO does not react with CO_2_ at the operating conditions of interest), its lower content in dolomites explains the lower position of the dolomite-related curves once compared to those for limestones.

**Figure 10:**
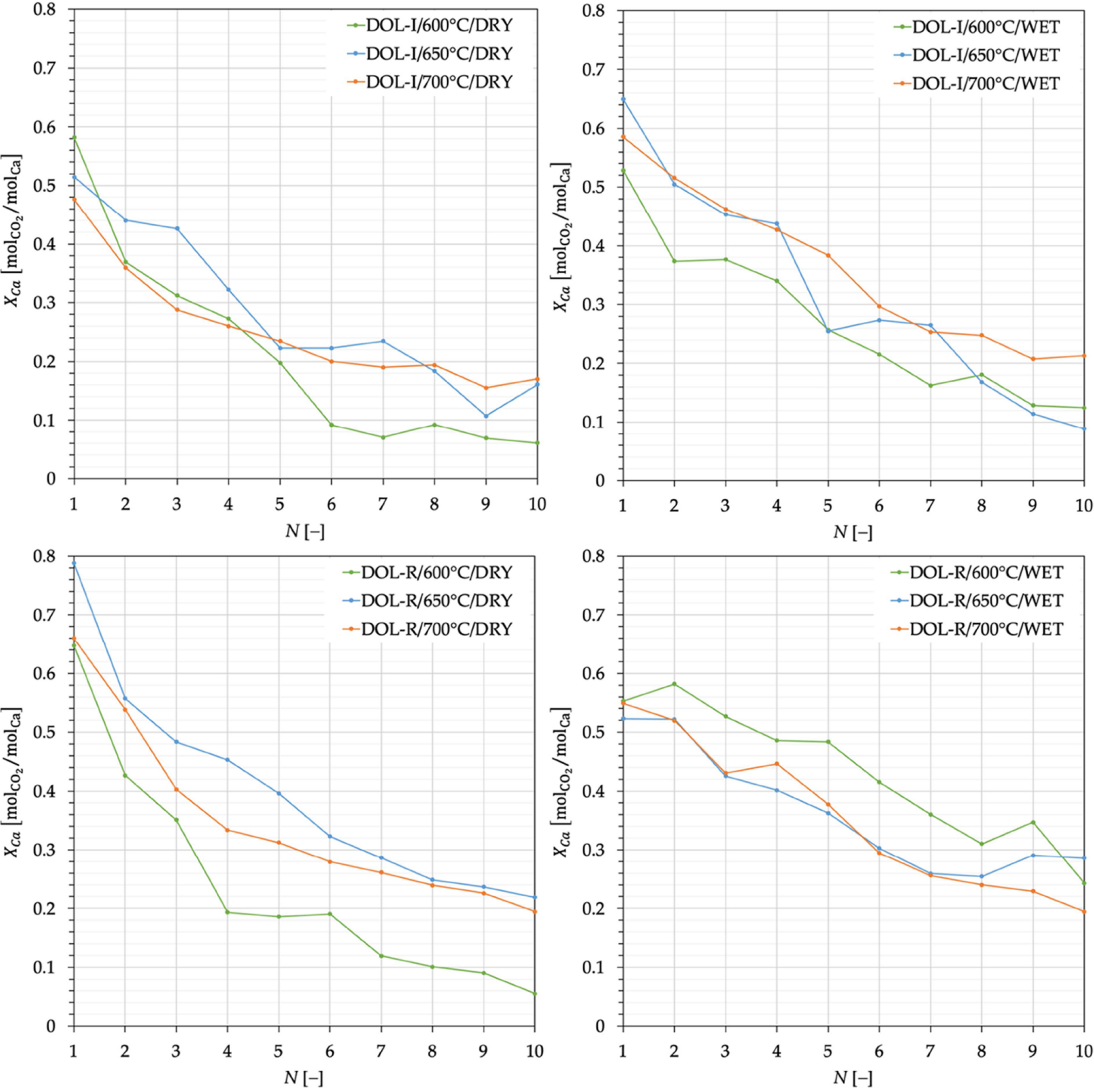
Degree of calcium carbonation, eq 2, for DOL-I and DOL-R as a function of the number of carbonation stages carried out at temperatures of 600, 650, and 700 °C, in the absence (dry conditions) and presence (wet conditions) of steam.

Therefore, an assessment concerning CaO reactivity should be performed in terms of *X*_Ca_ (eq 2). To this end, data in Figure 3 have been worked out in terms of the degree of Ca carbonation (Figure 10) and, then, also Figure 9(left) has been reworked to yield Figure 9(right). Interestingly, in terms of Ca conversion, the performance of DOL-I is well within the min-max range for limestones, while that of (the more reactive) DOL-R is comparable to the best values observed for limestones, witnessing at least a physical role for MgO in dolomites, helping in preserving particle porosity and, thus, the accessibility of CO_2_ gaseous molecules to CaO, as also observed in our previous work.^26^ More in detail, as highlighted in the literature,^28^ the physical role of the inert MgO skeleton can be related to its ability to preserve a nanocrystalline CaO structure, hindering aggregation/sintering of CaO grains. This favors CaO carbonation in the solid-state diffusion controlled phase, although the segregation of MgO, as long as the cycles proceed, makes its stabilizing role progressively less relevant.

It is interesting to note that the decreasing trend in capture capacity along the cycles shown in Figure 9 for the dolomites appears to be similar to that found for the 6 limestones, indicating a comparable effect of sintering during the tests. This result is different from what was observed under the (typically) more severe calcium looping conditions (in particular, calcination carried out at 940 °C in 70% CO_2_) in Coppola et al.,^26^ where a milder decrease trend was found for dolomite with respect to limestone, indicating a better resistance to sintering for the former. It is likely that such better resistance to sintering of dolomite, due to the presence of refractory MgO within the particle structure, becomes only evident when harsher operating conditions are used (especially during calcination), i.e., temperatures above 900 °C and high CO_2_ concentrations.

**Figure 11:**
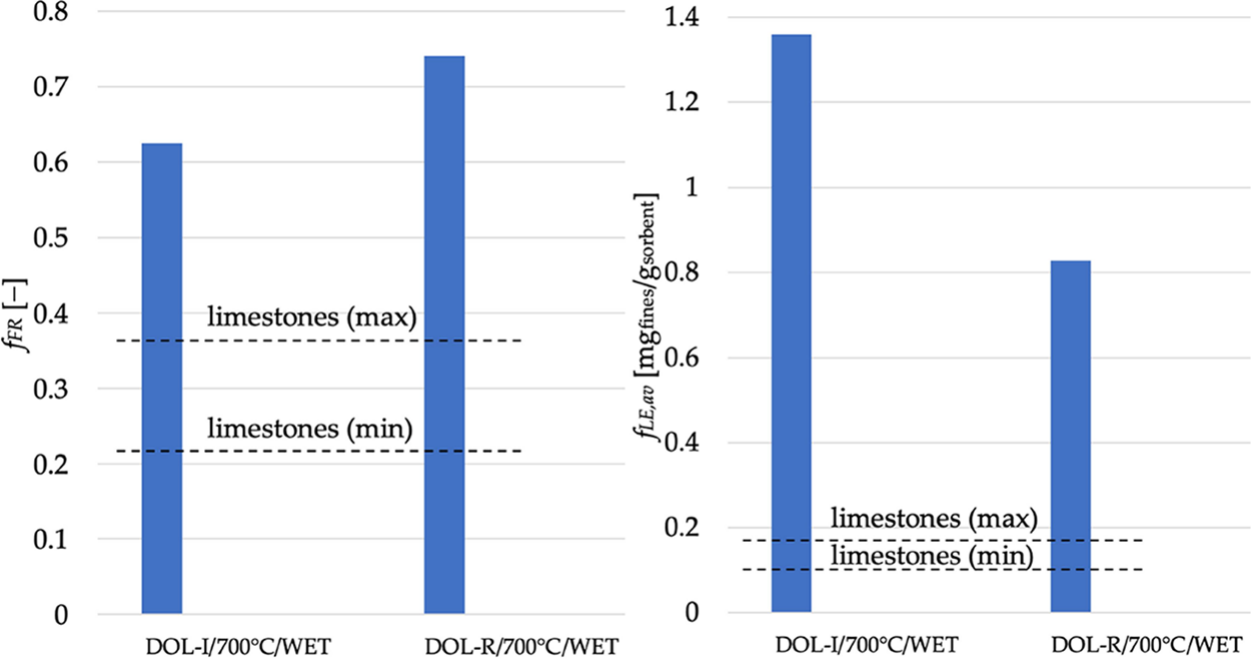
Left: cumulative mass fraction of fragments; Right: average value for the fraction of fines lost by elutriation, for DOL-I and DOL-R (carbonated under wet conditions at 700 °C), compared with the range of values obtained, under identical conditions, for a set of 6 limestones.

Fragmentation for dolomites was definitely more extensive than that for limestones. To give an example (Figure 11(left)), under completely comparable operating conditions, *f*_FR_ for the 6 different limestones (in the case of wet carbonation at 700 °C) was in the range 22–36%, while it was 62% and 74% for DOL-I and DOL-R, respectively. By regrouping our previous data obtained on the set of 6 limestone sorbents, and under equal operating conditions, it can be concluded that the fraction of fragments for dolomites increases by a factor of 2–2.5 vs limestones, given the lower mechanical resistance of MgO present in dolomites. It is, again, recalled that extensive in-bed fragmentation (with fragments remaining in the reactive system as nonelutriated particles) can help the sorbent behavior and can concur in explaining the very good degree of Ca carbonation observed, e.g., in Figure 9(right), for dolomites.

A similar picture emerges when comparing elutriation data in terms of fraction of fines lost by elutriation. Keeping the example of wet carbonation at 700 °C, Figure 11(right) illustrates that *f*_LE,*av*_ for the 6 different limestones was in the range 0.102–0.170 mg_fines_/g_sorbent_, while here it was 1.360 mg_fines_/g_sorbent_ and 0.828 mg_fines_/g_sorbent_ for DOL-I and DOL-R, respectively. By regrouping our previous data on the set of 6 limestone sorbents, the average value for the fraction of fines lost by elutriation increases (in the case of dolomites) by a factor from 2.7 up to 10 times compared to limestones. Apart from a more fragile structure for Mg-containing sorbents, we have already recalled that sorbents with higher CO_2_ capture capacity ξ (such as limestones) have a harder outer shell and are thus more resistant to surface wear.

## 4 Conclusions

The main information retrieved from this study, which can be of help for the design of a DIFB-SEG process for enhanced production of H_2_, is

1. Thermal sintering affects dolomites performance upon iterated cycles similarly to limestones. The loss of CO_2_ capture capacity between the first and the 10th cycles was in the range 45–92%. As reported in the literature,^29−31^ thermal pretreatments (precalcination) in a CO_2_-rich atmosphere may promote, for Ca-based sorbents (limestones, dolomites), CaO crystallization with the formation of a skeleton and a porous texture more stable toward sintering phenomena;
2. Steam is capable to positively influence the dolomites behavior, while the increase in carbonation temperature did not show a clear effect, due to counteracting (kinetic, thermodynamic, diffusive, sintering) phenomena;
3. Fragmentation of dolomites, expressed in terms of cumulative mass fraction of fragments after 10 cycles, was not lower than 59% (and as high as 80%), depending on the operating conditions. Fragmentation phenomena, indeed, can concur in increasing the CO_2_ capture capacity, provided that the fragments remain in the bed as nonelutriated particles;
4. The elutriation rate is (i) a function decreasing with the number of cycles (the decrease between the first and the 10th cycle is always >94%); (ii) markedly more relevant (by a 5–26 factor) upon calcination than carbonation; (iii) affected by the presence of steam, which makes the external particle structure more fragile; (iv) generally less relevant for sorbents with higher CO_2_ capture, a phenomenon that hardens the particles outer layer;
5. In comparison with limestones (nearly pure CaCO_3_), the presence of MgCO_3_ in dolomites, lowering the content of Ca, makes them less capable to capture CO_2_ (per unit mass of sorbent); however, by normalizing on the Ca content, the performance of dolomites is comparable to that of limestones;
6. Dolomites are much more prone to undergo fragmentation (the cumulative mass fraction of fragments after 10 cycles increases by a factor of 2–2.5) and elutriation (the average mass fraction of fines lost by elutriation increases by a factor of 2.7–10) than limestones, due to their intrinsic more fragile structure.

Concluding with a comment on the appropriateness of using dolomites or limestones as DIFB-SEG sorbents, on the laboratory scale and on the basis of our results, given the comparable degree of CaO conversion between limestones and dolomites, the greater tendency of dolomites to fragmentation and attrition makes them less attractive than limestones. On larger scales, the dolomites’ lower CO_2_ capture capacity also plays an important role, as the total amount of sorbent used must be considered (and not only that of CaO). Recently, the literature^15^ has proposed a techno-economic analysis on the topic, also comparing limestones and dolomites, confirming the preference for limestones. At the same time, there is literature^28^ that suggests the use of dolomites due to, for instance, a lower calcination temperature (i.e., lower energy penalty). It remains true, however, that there could be reasons of practical utility (in terms of availability and costs) that, at the plant site, could suggest the use of a dolomite. In this case, data on capture capacity and attrition/fragmentation tendency are important, since they contribute to the correct sizing of a DIFB-SEG plant (for example, to the calculation of the fresh sorbent make up stream to compensate for both the loss of reactivity of the material and the physical loss of fines by elutriation).
